# Influence of implantation of diffractive trifocal intraocular lenses on standard automated perimetry

**DOI:** 10.1186/s12886-022-02372-4

**Published:** 2022-04-02

**Authors:** Jinhee Lee, Yosai Mori, Keiichiro Minami, Kazunori Miyata

**Affiliations:** 1 Miyata Eye Hospital, Miyakonojyo, Miyazaki Japan; 2 Department of Ophthalmology, University of Tokyo, Tokyo, Japan

**Keywords:** Trifocal intraocular lens, Automated perimetry, Mean deviation, Foveal sensitivity

## Abstract

**Background:**

This prospective comparative study aimed to investigate the influence of diffractive trifocal intraocular lenses (IOLs) implantation on standard automated perimetry.

**Methods:**

Patients with no diseases affecting the visual field had undergone cataract surgery following the implantation of trifocal or monofocal IOLs from July 2019 to August 2020 were recruited. The normality of the anterior and posterior segments and absence of glaucomatous optic nerve cupping were confirmed preoperatively by slit-lamp examination. Standard automated perimetry was performed using Humphrey Visual Field 10–2 testing, 2–3 months after cataract surgery in only one eye per patient. The mean deviation (MD) and foveal sensitivity were compared between IOLs in eyes with acceptable reliability indices and best-corrected visual acuity of 20/25 or better.

**Results:**

Among the 83 eyes of the 83 patients included, 39 and 29 eyes eligible for perimetry analysis had trifocal and monofocal IOLs, respectively. The mean MD and foveal sensitivity in eyes with trifocal IOLs were significantly lower than those in eyes with monofocal IOLs (*P* < 0.021), with mean differences of 0.77 and 1.01 dB, respectively.

**Conclusion:**

The comparison in nonglaucomatous eyes demonstrated that the influence of trifocal IOLs on standard automated perimetry was greater than that of monofocal IOLs.

## Background

Various presbyopia-correcting intraocular lenses (IOLs) have been used clinically to reduce spectacle dependence and improve patient quality of life. In contrast, photic phenomena inherent in presbyopia-corrected IOLs are of concern. Additionally, degradation of visual field sensitivity has been observed with the use of diffractive bifocal and extended depth of focus (EDOF) IOLs [1–4]. Differences in mean deviation (MD) of 1.40—2.08 dB have been measured by standard automated perimetry (SAP) between monofocal and bifocal IOLs [1–4]. The influence of EDOF IOLs is comparable to that of monofocal IOLs [3, 4]. While it is still unknown why the influence varies with the type of diffractive multifocal IOL, the loss of light and disturbed point spread function due to diffractive optics are considered possible factors [5, 6].

Recently, trifocal IOLs have become available, enabling acceptable near, intermediate, and far visual acuity [7, 8], retaining visual acuities at far and near distances comparable to those of diffractive bifocal IOLs [9, 10]. As more complicated diffraction optics with four foci are used [5], PanOptix® trifocal IOLs (Alcon Laboratories, Fort Worth, TX) can induce an equal or greater influence on visual field sensitivity than bifocal IOLs. It is important to evaluate the degradation in the use of a diffractive trifocal IOL, as glaucoma is mostly a preoperative complications [11]. This prospective comparative study aimed to evaluate the influence of trifocal IOLs on the outcomes of SAP.

## Methods

### Participants

This prospective study was approved by the institutional review board of Miyata Eye Hospital and was performed in accordance with the tenets of the Declaration of Helsinki. Written informed consent was obtained after explaining the purpose and methods of the study to the patients. Patients who underwent cataract surgery with implantation of a diffractive trifocal IOL or monofocal IOL (TFNT00 and SN60WF, respectively; Alcon Laboratories) from July 2019 to August 2020 were recruited. The inclusion criteria were non-glaucomatous patients aged 20 years or older, axial length (AL) of 21—26 mm, intraocular pressure (IOP) < 21 mmHg, and no irregular astigmatism on the cornea, as described previously [4]. Non-glaucomatous conditions were confirmed with glaucoma diagnostic criteria used in previous population studies [12–15], that is, vertical cup-to-disc ratio > 0.7, rim width < 0.1-disc diameter, retinal nerve fiber layer defect, and disc hemorrhage. AL and IOP were measured using an OA-2000 optical biometer (Tomey Corporation, Nagoya, Japan) and an FT-1000 non-contact tonometer (Tomey), respectively. Corneal astigmatism was measured using an anterior segment optical coherence tomography CASIA 2 (Tomey) to examine for the absence of irregular astigmatism and astigmatism less than 1.00 D. Eyes with any history of disease that might affect the visual field, such as glaucoma, ocular surgery, or trauma, were excluded.

The participants were divided into two groups according to the implanted IOL: monofocal SN60WF (monofocal group), and diffractive trifocal TFNT00 (trifocal group). The number of eyes in each group was 11 or more. This minimum sample size was required for detecting a difference in MD values of 1.43 dB between diffractive bifocal and monofocal IOLs in our previous report, which was conducted under the same conditions in visual field testing as in the present study [4], according to a t-test with a significance level of 0.05 and a detection power of 0.8, with the effect size of 1.3.

### Intraocular lenses

The implanted IOLs were one-piece hydrophobic lenses that used the same platform with a total length of 13 mm and an optic diameter of 6 mm. Diffractive optics of the multifocal TFNT00 with added powers of 2.17 and 3.25 D were used for intermediate and near vision, respectively.

Before surgery, the IOL type was chosen according to the patient’s preference for postoperative vision. For patients preferring far and near vision with no or less spectacle use, multifocal TFNT00 was recommended. For patients who were not interested in presbyopia correction or were uncomfortable with the photic symptoms associated with the use of TFNT00, monofocal SN60WF was recommended. With sufficient explanation of the benefits and risks of both types of IOLs, the choices of implanted IOLs were determined.

After topical anesthesia, the cataract was removed using a continuous curvilinear capsulorrhexis and phacoemulsification technique through a 2.2-mm superior sclerocorneal incision. IOLs were implanted in capsular bags using injectors.

### Postoperative examination

Two to three months after surgery, best-corrected visual acuity (BCVA) and SAP were examined in the same manner as previously described [4]. All participates underwent fundus examination using mydriatic agents and were confirmed to have no nerve fiber layer defects. The SAP was measured using a Humphrey Field Analyzer (Carl Zeiss Meditec, Dublin, CA, USA) with the SITA standard threshold test algorithm under a 10–2 grid after correcting the refractions for the testing distance (33 cm), white stimulus color, Goldmann size III target, and a background luminance of 31.5 apostilbs. SAP results were judged to be reliable when a fixation loss rate lower than 15%, a false-positive rate lower than 15%, a false-negative rate lower than 20%, and pupil size larger than 2.5 mm were obtained. The influence on SAP was evaluated using indices of MD and foveal sensitivity. Foveal sensitivity was obtained as the foveal threshold in SAP measurements [16].

### Statistical analysis

If the postoperative BCVA was worse than 20/25 (equivalent to 0.10 logMAR) or the reliability of the SAP result was not verified, the eyes were excluded from further analysis. The right eye was included in the analysis when both eyes were eligible. Shapiro–Wilk tests were performed to confirm the normality of the demographic data and perimetry results. For parameters exhibiting a normal distribution, the t-test was used for comparison. Otherwise, the Mann–Whitney U test was used. Statistical analyses were performed using R version 3.5.2 (The R Foundation for Statistical Computing, Vienna, Austria). Statistically significance was set at *P* < 0.05.

## Results

A total of 83 eyes of 83 patients, including 47 eyes with trifocal IOLs and 36 eyes with monofocal IOLs were assessed. No complications occurred during any of the surgeries. Due to insufficient reliability of the SAP measurements, 15 eyes (trifocal, eight eyes; monofocal, seven eyes) were excluded from the analysis. Thus, 39 and 29 eyes with trifocal and monofocal IOLs, respectively, were used for analysis. Shapiro–Wilk tests showed that age, pupil size, AL, IOP, and MD followed a normal distribution, while BCVA and foveal sensitivity did not follow a normal distribution. Table 1 shows the demographic data and BCVA values of both groups. The BCVA in the monofocal group was significantly better than that in the trifocal group (*P* = 0.042), while the mean difference of 0.03 logMAR was clinically negligible.

**Table 1:** Demographic data and postoperative visual acuity in eyes with trifocal and monofocal intraocular lenses

Intraocular lens	Trifocal	Monofocal	*P* value
Eyes	39	29	
Male/Female	18/21	8/21	
Right / Left eye	23/16	17/12	
Age, years	67.1 ± 7.0 [53 to 80]	67.8 ± 5.5 [56 to 83]	0.62^*^
Pupil size, mm	5.0 ± 1.1 [3.0 to 8.4]	4.8 ± 1.0 [2.7 to 6.5]	0.57^*^
Axial length, mm	23.8 ± 1.1 [21.29 to 25.9]	23.7 ± 0.9 [21.97 to 25.46]	0.71^*^
Intraocular pressure, mmHg	14.4 ± 3.0 [11 to 20]	14.2 ± 2.5 [8 to 18]	0.70^*^
BCVA, logMAR	-0.11 ± 0.07 [-0.18 to 0.10] Median: -0.08	-0.14 ± 0.06 [-0.18 to -0.05] Median: -0.18	0.042^**^

mean ± standard deviation [range]

^*^t-test

^**^Mann–Whitney U test; BCVA: best-corrected visual acuity

**Fig. 1:**
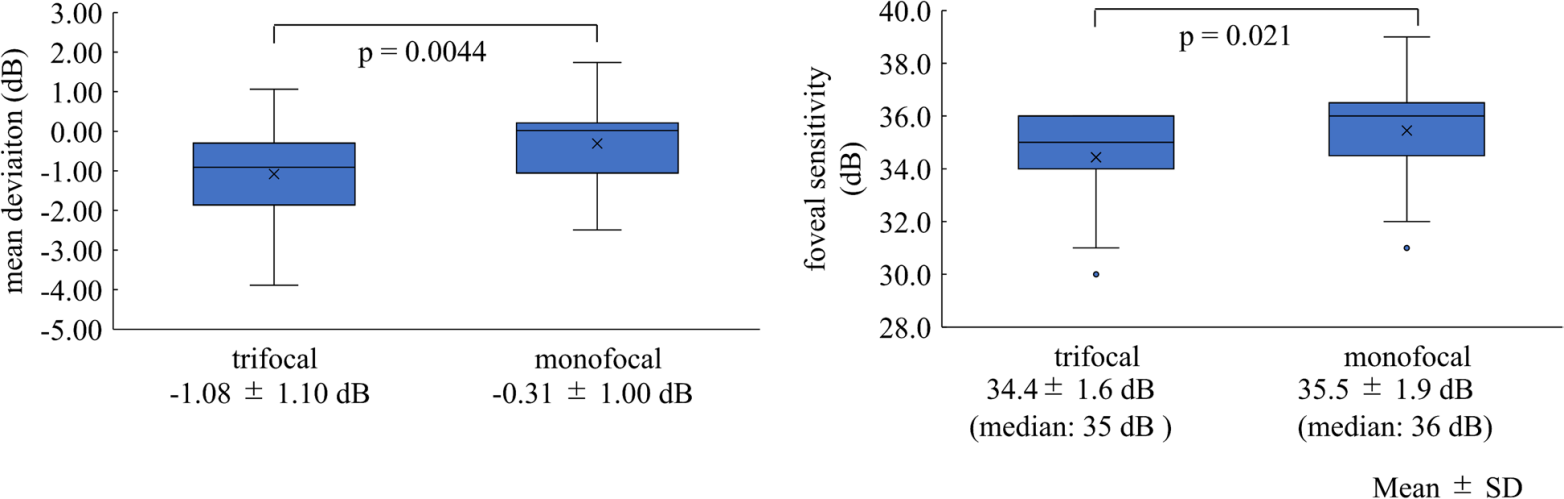
Box plots of mean deviation (left) and foveal sensitivity (right) for 10–2 grid standard automated perimetry in eyes with trifocal and monofocal intraocular lenses (IOLs) in the current study. The mean deviation and foveal sensitivity with monofocal IOLs are significantly higher than those with trifocal IOLs (*P* < 0.021)

Figure 1 shows box plots of MD and foveal sensitivity. The mean MD and median foveal sensitivity of the trifocal group were -1.08 and 35 dB, respectively, and were significantly lower than those of the monofocal group (*P* < 0.021). Differences in the mean MD and median foveal sensitivity were 0.77 and 1 dB, respectively. None of the eyes demonstrated relevant pathological characteristics in the SAP results.

## Discussions

**Fig. 2:**
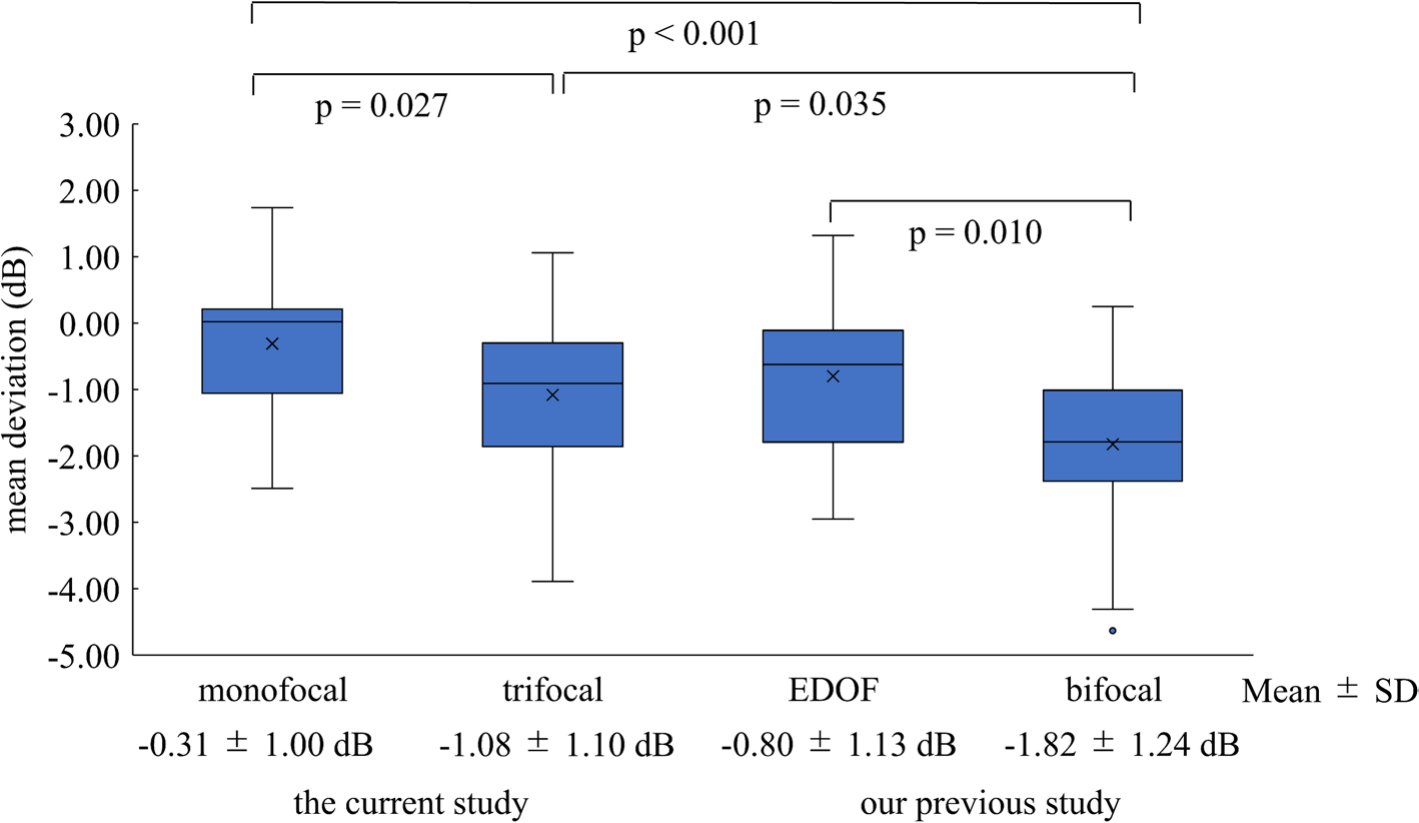
Box plot of mean deviation for 10–2 grid standard automated perimetry in eyes with monofocal, diffractive trifocal, extended depth of focus (EDOF), and bifocal intraocular lenses (IOLs) obtained in previous [4] and current studies. The mean deviation values associated with the use of the trifocal IOLs are lower than those for the use of the monofocal IOLs (*P* = 0.027, t-test with Holm’s multiple comparisons), not different from those for the use of EDOF IOLs, and higher than those for the use of bifocal IOLs (*P* = 0.035)

The current comparison between 39 and 29 eyes of the trifocal and monofocal groups, respectively, revealed the influence of diffractive trifocal IOLs on postoperative visual field sensitivity. The trifocal group exhibited a significantly lower MD. Previous comparisons showed significant reductions in the use of diffractive bifocal IOLs [1–4], and the differences ranged from 1.40 to 2.08 dB. Conversely, the difference from monofocal IOL was shown to be 0.26 D in the use of EDOF IOLs [3], although the study design and SAP setting were different. Because our previous comparison of monofocal, bifocal, and EDOF IOLs used the same protocol except for the implanted IOLs [4], we compared the MD values in the previous and current results. Figure 2 shows the MD values in the use of monofocal (SN60WF), diffractive trifocal (TFNT00), EDOF (ZXR00V, Johnson & Johnson Surgical Vision, Santa Ana, CA), and bifocal (ZMB00, Johnson & Johnson Surgical Vision) IOLs. Under 10–2 testing, the MD values associated with the use of trifocal IOLs were better than those for the use of bifocal IOLs and worse than those for monofocal IOLs (*P* = 0.035 and 0.027, respectively, t-test with Holm’s multiple comparisons). No significant differences were found in EDOF IOLs. Although the designs of diffractive optics were different, it was speculated that the difference in energy loss in multifocal IOLs, such as 20% for ZMB00, 12% for TFNT00, and 8% for ZXR00V, would contribute to the SAP results [6, 17]. Further investigation is necessary to identify the underlying etiology.

In the current study, the foveal sensitivity of eyes with trifocal IOLs was lower than that of the eyes with monofocal IOLs. Takahashi et al. reported a 3.0 dB significant difference in foveal sensitivity between eyes with bifocal and monofocal IOLs [3]. In the previous comparison, such a significant difference was not detected [4], whereas the mean difference (1.32 dB) was larger than in the current results. Compared to this, the current results were obtained from a larger sample size and comparison of the two groups, which would increase the detection power.

This study had some limitations. First, visual field testing was performed only once. The results of SAP are variable, even in healthy participates [18]. Hence, we verified the examination using reliability measures, such as fixation loss, false-positive, and false-negative. Second, the mean sensitivity was not evaluated. In our previous comparison of EDOF, bifocal, and monofocal IOLs, the mean sensitivity was calculated and analyzed [4]. As there was no significant difference in age between the trifocal and monofocal IOL groups, the mean sensitivity was expected to be similar to that of MD [19]. Finally, only 10–2 grid SAP were examined, while SAP with 24–2 grid or 30–2 grid were conventionally examined. Previous studies have shown that there is no difference between the results under 30–2 and 10–2 grids [1–4]. In contrast, variability increased with visual field eccentricity in SAP in normal participates [20]; therefore, we concluded to use the 10–2 grid.

## Conclusion

The influence of trifocal IOLs on SAP was greater than that of monofocal IOLs in healthy participates.

## Data Availability

The datasets used and/or analyzed during the current study are available from the corresponding author upon reasonable request.

## Abbreviations

IOL: Intraocular Lens
EDOF: Extended Depth-Of-Focus
MD: Mean Deviation
SAP: Standard Automated Perimetry
AL: Axial Length
IOP: Intraocular Pressure
BCVA: Best-Corrected Visual Acuity

## References

[1] Farid M, Chak G, Garg S, Steinert RF (2014). Reduction in mean deviation values in automated perimetry in eyes with multifocal compared to monofocal intraocular lens implants. Am J Ophthalmol.

[2] Aychoua N, Junoy Montolio FG, Jansonius NM (2013). Influence of multifocal intraocular lenses on standard automated perimetry test results. JAMA Ophthalmol.

[3] Takahashi M, Yamashiro C, Yoshimoto T, Kobayashi Y, Higashijima F, Kobayashi M (2020). Influence of extended depth of focus intraocular lenses on visual field sensitivity. PLoS One..

[4] Lee J, Mori Y, Nejima R, Minami K, Miyata K (2020). Influence of implantations of extended depth-of-focus on standard automated perimetry. Sci Rep.

[5] Yoo YS, Whang WJ, Byun YS, Piao JJ, Kim DY, Joo CK (2018). Through-Focus Optical Bench Performance of Extended Depth-of-Focus and Bifocal Intraocular Lenses Compared to a Monofocal Lens. J Refract Surg.

[6] Percival SP (1989). Prospective study of the new diffractive bifocal intraocular lens. Eye (Lond).

[7] Gatinel D, Loicq J (2016). Clinically Relevant Optical Properties of Bifocal, Trifocal, and Extended Depth of Focus Intraocular Lenses. J Refract Surg.

[8] Sudhir RR, Dey A, Bhattacharrya S, Bahulayan A (2019). AcrySof IQ PanOptix Intraocular Lens Versus Extended Depth of Focus Intraocular Lens and Trifocal Intraocular Lens: A Clinical Overview. Asia Pac J Ophthalmol (Phila).

[9] Shen Z, Lin Y, Zhu Y, Liu X, Yan J, Yao K (2017). Clinical comparison of patient outcomes following implantation of trifocal or bifocal intraocular lenses: a systematic review and meta-analysis. Sci Rep.

[10] Zamora-de La Cruz D, Zúñiga-Posselt K, Bartlett J, Gutierrez M, Abariga SA (2020). Trifocal intraocular lenses versus bifocal intraocular lenses after cataract extraction among participants with presbyopia. Cochrane Database Syst Rev.

[11] Negishi K, Hayashi K, Kamiya K, Sato M, Bissen-Miyajima H (2019). Survey Working Group of the Japanese Society of Cataract and Refractive Surgery. Nationwide Prospective Cohort Study on Cataract Surgery With Multifocal Intraocular Lens Implantation in Japan. Am J Ophthalmol..

[12] Foster PJ, Buhrmann R, Quigley HA, Johnson GJ (2002). The definition and classification of glaucoma in prevalence surveys. Br J Ophthalmol.

[13] Iwase A, Suzuki Y, Araie M, Yamamoto T, Abe H, Shirato S (2004). The prevalence of primary open-angle glaucoma in Japanese: the Tajimi Study. Ophthalmology.

[14] He M, Foster PJ, Ge J, Huang W, Zheng Y, Friedman DS (2006). Prevalence and clinical characteristics of glaucoma in adult Chinese: a population-based study in Liwan District. Guangzhou Invest Ophthalmol Vis Sci.

[15] Topouzis F, Wilson MR, Harris A, Anastasopoulos E, Yu F, Mavroudis L (2007). Prevalence of open angle glaucoma in Greece: the Thessaloniki Eye Study. Am J Ophthalmol.

[16] Chiba N, Imasawa M, Goto T, Imai M, Iijima H (2012). Foveal sensitivity and visual acuity in macular thickening disorders. Jpn J Ophthalmol.

[17] Gundersen KG, Potvin R (2017). Trifocal intraocular lenses: a comparison of the visual performance and quality of vision provided by two different lens designs. Clin Ophthalmol.

[18] Wall M, Doyle CK, Zamba KD, Artes P, Johnson CA (2013). The repeatability of mean defect with size III and size V standard automated perimetry. Invest Ophthalmol Vis Sci.

[19] Monsalve B, Ferreras A, Calvo P, Urcola JA, Figus M, Monsalve J (2017). Diagnostic ability of Humphrey perimetry, Octopus perimetry, and optical coherence tomography for glaucomatous optic neuropathy. Eye (Lond).

[20] Chauhan BC, Johnson CA (1999). Test-retest variability of frequency-doubling perimetry and conventional perimetry in glaucoma patients and normal subjects. Invest Ophthalmol Vis Sci.

